# Extraction of Carotenoids from Tomato Pomace via Water-Induced Hydrocolloidal Complexation

**DOI:** 10.3390/biom10071019

**Published:** 2020-07-09

**Authors:** Jayesree Nagarajan, Hang Pui Kay, Nagendra Prasad Krishnamurthy, Nagasundara Ramanan Ramakrishnan, Turki M. S. Aldawoud, Charis M. Galanakis, Ooi Chien Wei

**Affiliations:** 1 Chemical Engineering Discipline, School of Engineering, Monash University Malaysia, Bandar Sunway, Selangor 47500, Malaysia; jayzeesmile@gmail.com (J.N.); cindyhpk@gmail.com (H.P.K.); ramanan@monash.edu (N.R.R.); 2 World Pranic Healing Foundation, India Research Center, 4th Main, Saraswathipuram, Mysore, Karnataka 570009, India; knag76@gmail.com; 3 College of Science, King Saud University, Riyadh 11451, Saudi Arabia; tdawoud@ksu.edu.sa; 4 Research & Innovation Department, Galanakis Laboratories, 73131 Chania, Greece; 5 Food Waste Recovery Group, ISEKI Food Association, 1190 Vienna, Austria; 6 Monash-Industry Palm Oil Education and Research Platform (MIPO), Monash University Malaysia, Bandar Sunway, Selangor 47500, Malaysia

**Keywords:** carotenoid–pectin complexation, tomato pomace, pectin, lycopene, antioxidant

## Abstract

Agro-industrial waste is a largely untapped natural resource of bioactive compounds including carotenoids and pectin. However, conventional solvent extraction involves the excessive use of organic solvents, costly equipment, and tedious operation. These limitations of conventional extraction methods could be prospectively overcome by the carotenoid–pectin hydrocolloidal complexation. The complexation of lycopene and pectin was efficiently promoted in an aqueous environment, resulting in the colloidal complexes that can be subsequently recovered by sedimentation or centrifugation. In this study, the potential of carotenoid–pectin complexation on tomato pomace containing carotenoids and pectin was evaluated. Tomato pomace is a rich source of lycopene, β-carotene as well as pectin, making it suitable as the raw material for the carotenoid extraction. The extraction of carotenoid and pectin from tomato pomace was optimized using response surface methodology. The maximum recovery was 9.43 mg carotenoid fractions/100 g tomato pomace, while the purity of carotenoid-rich fractions was 92%. The antioxidant capacity of carotenoids extracted from the complexation method was found to be higher than that from the solvent extraction method. Moreover, extraction yield and antioxidant capacity of carotenoid obtained from the carotenoid–pectin complexation were comparable to that from solvent extraction. The carotenoid–pectin complexation is a promising green approach to valorize agro by-products for the extraction of valuable carotenoids.

## 1. Introduction

Currently, food bioactives are widely considered as important compounds in supporting human’s immune system, especially in the era of the COVID-19 pandemic [1]. On the other hand, the food-processing sector generates a massive volume of by-products, which have been well recognized as the invaluable and natural sources of bioactive compounds, dietary fibers and antioxidants. For example, juice extraction could yield 5.5 million metric ton (MMT) of waste including pomace, while the canning and frozen food industries generate around 6 MMT of plant-derived waste annually [2]. Interestingly, the amounts of bioactives found in some agricultural by-products (e.g., pulp, peel, and seed) are even greater than that in the edible part of the fruit [3]. The reutilization of these agri-food wastes for the recovery of bioactive compounds is efficient in reducing the disposal of organic waste from food processing. Carotenoids are a class of antioxidant compounds that exists widely in nature, especially in all colored fruits, vegetables, and flowers; they have important health effects, e.g., exert preventive activity against chronic diseases. The estimated value of the global market of carotenoids in 2017 was 1.5 billion USD, and the value was forecasted to reach 2.0 billion USD in 2022 [4].

Tomato products are a popular global commodity as their production exceeded 210 MMT in 2012 [5]. The major bioactive compound in tomato is lycopene, which constitutes 80–90% of the total carotenoids [6]. Other carotenoids present in the tomato include β-carotene, phytoene, phytofluene, neurosporene, and lutein [6]. The lycopene content in tomato is mainly associated to the variety of tomato and the applied extraction process. Similar to lycopene, β-carotene is another noteworthy group of carotenoids popularly associated with its antioxidant property. This carotenoid is responsible for the reddish-orange appearance of many crops.

The recovery of compounds from food processing by-products is usually conducted using the 5-Stage Universal Recovery Process: pre-treatment, separation of macro- and micro-molecules, extraction, purification, and product formation [7]. Among these stages, the extraction is the most essential step [8]. Conventionally, organic solvents (e.g., hexane, acetone, methanol, and ethanol) and solvent combinations have been used in the solvent extraction of carotenoids [9]. Solvent selection is crucial and largely dependent on the polarity of carotenoids. A mixture of hexane, ethanol, and acetone is usually adopted for the extraction of polar and non-polar carotenoids. The extraction from tomato processing waste (i.e., skin and seeds of tomato) was enhanced by the accelerated solvent extraction [10], which increased the permeability of cells and improved the metabolite diffusion via structural changes in the cell membrane; high-pressure-assisted extraction of carotenoid at 7000 bar reduced the processing time and the consumption of solvents [10]. In addition, the pretreatment of tomato waste by enzymes increased the yield of lycopene up to 10 folds in a solvent extraction based on ethyl lactate [10]. Enzyme-assisted extraction uses the hydrolytic enzymes (e.g., pectinase and cellulose) to break down the structure of cell walls, thereby exposing the intracellular materials and facilitating their diffusion [9]. In another study [11], extraction at 100 °C for 6.5 h yielded 88% of lycopene from tomato wastes.

Most of the conventional solvent extraction methods have drawbacks that are related to the long extraction times and the consumption of a great amount of organic solvents [12]. The utilization of large volumes of organic solvents during the extraction process raises concerns on the environmental and health hazards [13]. To the best of our knowledge, the studies dealing with the recovery of carotenoids from natural resources and food processing by-products without the use of organic solvents are limited. Recently, our research group reported a green extraction method that relies on a simple water-induced complexation of lycopene and pectin [14]. This method has been successfully applied for the extraction of both pectin and lycopene from pink guava decanter. The formation of a hydrocolloidal system was believed to be facilitated by pectin in the presence of carotenoids in aqueous solution. The complexation of carotenoid and pectin was governed by the extraction parameters including pH, solid loading, temperature, and stirring conditions. The applicability of this facile extraction method can be extended to fruit-processing wastes that are naturally abundant in pectin and carotenoids.

The aim of the current study was to further explore the extraction of carotenoids from tomato pomace using the recently developed water-induced hydrocolloidal complexation approach. The carotenoid and pectin recovered from the complexation process were structurally analyzed by high-performance liquid chromatography (HPLC) and spectroscopy. The influential extraction parameters were identified through factorial screening prior to the optimization of extraction yield. The fractionated carotenoids were then compared in terms of yield, purity, and antioxidant capacity with the carotenoids extracted using the optimized conventional solvent extraction.

## 2. Materials and Methods

### 2.1. Materials

HPLC grade solvents, including methanol, 2-propanol, and tetrahydrofuran (THF) were purchased from Merck (Darmstadt, Germany). Analytical grade solvents, including acetone, *n*-hexane, ethanol, methanol, and triethylamine, were also purchased from Merck (Darmstadt, Germany). Potassium persulfate, 2,2-diphenyl-1-picrylhydrazyl (DPPH), 2,2′-azino-bis (3-ethylbenzthiazoline-6-sulfonic acid) (ABTS), dextran standard, pectin standard (from citrus peel), sodium chloride (NaCl), β-carotene standard, and lycopene standard (sum of isomers in ≥95% of corn oil) were purchased from Sigma Aldrich (St. Louis, MO, USA). The fresh tomatoes were purchased from the local market (Selangor, Malaysia).

### 2.2. Sample Preparation

The tomatoes were first washed with clean water and sliced into four equal sizes. After removing the seeds, the sliced tomatoes were blended using a juice extractor (model HR-1823, Philips). The leftover fibrous solid content was collected and used as the wet tomato pomace. For experiments based on the conventional solvent extraction method [15], the dried samples were used; briefly, the wet processed samples were oven-dried at 40 °C for 5 h to minimize the water content, before being crushed, ground, and separated using a 300-µm sieve. The moisture content in the tomato pomace was 89% (w/w). All the samples were stored in dark condition at −20 °C to prevent the degradation of carotenoid.

### 2.3. Conventional Extraction of Lycopene and β-Carotene from Tomato Pomace

The extraction protocol as reported by Kehili and co-workers [15] was adopted as the conventional method for the extraction of lycopene and β-carotene from tomato pomace. About 5 g of the dried tomato pomace was mixed at 200 rpm in 100 mL of hexane solution overnight at room temperature. The extract was filtered using Whatman cellulose filter paper (Grade 1; pore size = 11 µm) and the collected solid waste was re-extracted twice using 100 mL of fresh hexane under the same conditions. The hexane extract was combined and evaporated using a rotary evaporator at 40 °C. The concentrated carotenoid was dissolved with 2 mL of THF prior to the analysis.

### 2.4. Carotenoid–Pectin Complexation Method

The processed wet samples were used in the water–induced complexation of carotenoid and pectin. The sample was first loaded into the beaker containing 50 mL of water. The mixture was stirred using an overhead stirrer (model RW 20, IKA, Germany) for a defined incubation period. The variables of the experiments included pH, solid loading, stirring time, stirring speed, and incubation time. The mixture was then centrifuged to remove the heavier solid debris. Subsequently, the supernatant was subjected to a second round of centrifugation to separate the carotenoid–pectin complex. After decanting the solution, the wet carotenoid–pectin complex was recovered and weighted. Since the densities of the processed samples and the colloidal complex were different, the operations of centrifugation were optimized for the complete separation of plant debris and colloidal complex. The separation of solid debris was conducted by centrifuging the samples at 7500 rpm for 20 min, whereas the recovery of the colloidal complex was achieved by centrifuging the samples at 10,000 rpm for 5 min. In this extraction process, the presence of seeds in the tomato pomace altered the density of the sample and demanded more rounds of centrifugation. Hence, the processed tomato pomace used in this study was free of seeds.

The influential extraction parameters were first identified via experiments based on a two-level half factorial design, as shown in Table 1. The factorial screening comprised 19 runs of experiments, including the repetition of three central points that were in the intermediate ranges of variables. The total volume of extraction was 50 mL. The mass of the carotenoid–pectin complex (mg/100 g) was recorded as the response. The yields of the carotenoid–pectin complexes from tomato pomace were optimized using a response surface methodology. Based on the factorial screening results, the three most influential extraction parameters were used in 20 sets of experiments as per central composite design (CCD). The mass of the carotenoid–pectin complex extracted as well as the carotenoid content were determined as the responses. By using CCD, the statistically significant operating parameters were analyzed to determine the interactive effects between the variables on the tested responses. The significance of each variable was determined by *F* and *p* values, where a higher *F* value and a lower *p* value often exhibit a significant effect on the response [16]. Based on the recommended extraction conditions, each extraction model was further validated by comparing the experimental data with the predicted values.

### 2.5. Quantification and Structural Confirmation of Carotenoid and Pectin

The wet carotenoid–pectin complex was recovered and weighed gravimetrically. The complex was then suspended in 1 mL of THF. The pectin was removed by a round of centrifugation at 4500 rpm for 10 min, and the THF liquid fraction containing carotenoid suspended in THF was collected for further analysis. The absorbance of the sample was measured using a spectrophotometer (Genesys 20, Thermo Scientific, Waltham, MA, USA) at 480 nm, and the concentration of carotenoid was calculated using the calibration curves prepared by β-carotene standard and lycopene standard in the concentration range of 7.81–1000 ug/mL. The results were expressed as total carotenoid content (mg/100 g wet sample). The purity of carotenoid was analyzed using an HPLC system (Agilent Technologies 1200) equipped with a C30 reversed-phase column (250 mm × 46 ID, 5 µm, Waters, Zellik, Belgium) and a diode array detector. An isocratic elution was conducted using a mobile phase prepared by mixing methanol, isopropyl alcohol, and THF at a ratio of 30:30:35 [17]. The column temperature was kept at 35 °C and the injection volume was 10 µl. The flow rate through the column was set at 0.5 mL/min. The extracted pectin was structurally confirmed using Fourier-transform infrared spectroscopy (FTIR). An FTIR spectrometer (Nicolet iS10, Thermo Scientific) equipped with an attenuated total reflectance sampling accessory was used. The FTIR spectra of the fractionated pectin were obtained via a wavelength scan (550 cm^−1^ to 4000 cm^−1^) with a spectral resolution of 4 cm^−1^ in 64 scans.

### 2.6. Antioxidant Capacity of Carotenoid

The antioxidant capacity of the carotenoid fractions was measured using DPPH and ABTS free radical assays. Both assays quantitate the antioxidant potential of a sample based on the scavenging activities of carotenoids on the free radicals (DPPH or ABTS) added in the solution. The DPPH assay was conducted according to a previously reported method with some modifications [18]. Firstly, 100 μL of the sample solution was added to 900 μL of DPPH solution (0.2 mM; prepared in methanol). The mixture was thoroughly mixed before being subjected to incubation in dark condition for 20 min. The DPPH radical scavenging activity was determined using a spectrophotometer by measuring the absorbance at 517 nm. A solution of methanol and THF (900:100 μL) was used as the blank.

The ABTS assay was conducted based on a previously reported method [15] with some modifications. ABTS stock solution was prepared by mixing equal volumes of 7 mM ABTS solution and 4.9 mM of potassium persulfate solution. The mixture was kept in dark condition for 12–16 h. The ABTS stock solution was diluted with ethanol to reach an absorbance of 0.7 at 734 nm. After adding 100 μL of the sample solution to 900 μL of the diluted ABTS solution, the mixture was mixed by vortexing for 45 s. The ABTS radical scavenging activity was determined by measuring the absorbance at 734 nm. A solution of ethanol and THF (900:100 μL) was used as the blank.

For both assays, the percentage of scavenging activity was calculated using Equation (1):

(1) $$\mathrm{DPPH}\;\mathrm{or}\;\mathrm{ABTS}\;\mathrm{radical}\;\mathrm{scavenging}\;\mathrm{activity}\;\left(\%\right)=\frac{A_{i}-A}{A_{i}}\times 100$$

where *A*_i_ is the absorbance value of control solution (1 mL of DPPH or ABTS solution) and *A* is the absorbance value of mixture (DPPH or ABTS solution containing the sample) incubated for a defined period. The concentrations of DPPH or ABTS needed for decreasing the initial concentration of DPPH or ABTS radical concentration by 50% (IC_50_) were determined.

### 2.7. Statistical Analysis

The statistical experimental design was generated and analyzed using Design-Expert (Version 7.0, Stat-Ease Inc., Minneapolis, MN, USA). The interactions between variables and the effect of variables on the product recovery were statistically analyzed by analysis of variance (ANOVA). The value of *p* < 0.05 was regarded as statistically significant at a confidence level of 95%. The fitting accuracy of each model was analyzed based on the regression coefficient (*R*^2^) value. The analysis of the interaction between variables was performed using the three-dimensional (3D) response surface plots. The experimental data were reported as the mean values of triplicate measurements.

## 3. Results and Discussion

### 3.1. Structural Confirmation of Carotenoid and Pectin

**Figure 1:**
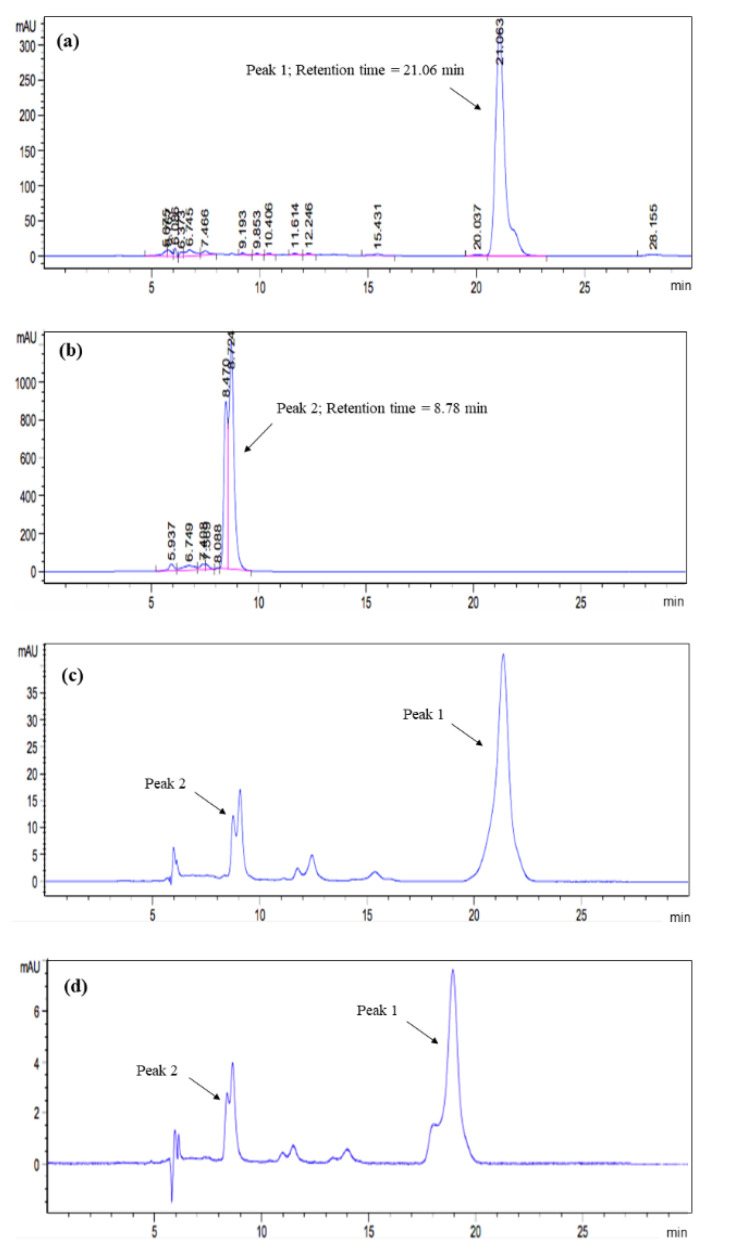
HPLC analyses of (**a**) standard lycopene, (**b**) standard β-carotene, and the samples extracted from tomato pomace using (**c**) solvent extraction, and (**d**) carotenoid–pectin complexation.

The carotenoid and pectin as extracted from tomato pomace using solvent extraction and carotenoid–complexation were structurally confirmed using HPLC and FTIR analyses. The HPLC chromatograms of the extracted carotenoids are shown in Figure 1.

The presence of lycopene and β-carotene in the crude extracts was confirmed by the peaks found in the chromatograms. Figure 1a,b show the HPLC peak of standard lycopene (peak 1; retention time = 21.06 min) and standard β-carotene (peak 2; retention time = 8.78 min) in 30 min of run time. For all the tested samples, the selectivity of carotenoid obtained from both solvent extraction and carotenoid–pectin complexation was identical. The extract of tomato pomace contained both lycopene and β-carotene. To evaluate the adequacy of the extraction method, the purity of carotenoid fractions was calculated by using the peak normalization method. The purity level of lycopene and β-carotene extracted using solvent extraction (Figure 1c) was 91%, which is slightly lower than that using complexation (92%, see Figure 1d). The minor unknown peaks found in the time range of 12–15 min (Figure 1c,d) affected the purity of sample. Although these minor peaks matched the peaks representing *cis*-lycopene isomers as described previously [17], they were not considered in this calculation as their retention time deviated far from the main peak representing *trans*-lycopene. Nonetheless, the complexation method can be concluded to be as good as solvent extraction, as both methods yielded carotenoid extract at a comparable level of purity. The broad peak at 8.9 min in the fraction from both extraction methods (Figure 1d) indicates the presence of β-carotene in both forms of *trans* and *cis* isomers [19]. Due to the overlapping peaks, the β-carotenes peaks were considered as a whole single peak.

**Figure 2:**
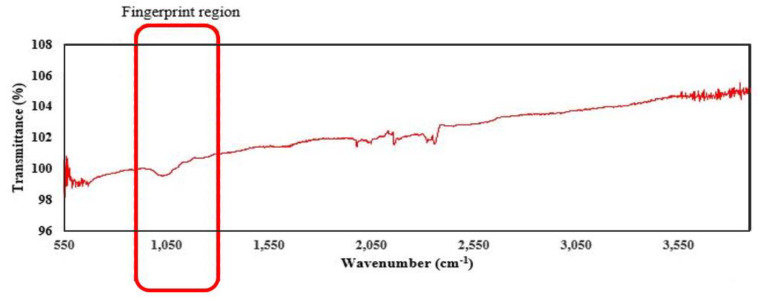
FTIR spectra of pectin recovered from tomato pomace.

The FTIR spectra of the air-dried pectin recovered from the extraction processes are shown in Figure 2 The wavelength range of 950–1200 cm^−1^ in the spectra is the “fingerprint” region of carbohydrates, confirming the major functional group in the polysaccharides [20]. Since pectin is a complex polysaccharide, the functional groups in its structure may vary, and the properties are governed by other factors. Apart from the C–O stretching, the FTIR spectrum of tomato pomace shows small peaks in the region between 2050–2500 cm^−1^, which corresponds to the C≡C group (lipids or fatty acids). This may indicate the traces of lycopene left during the fractionation procedure [21].

### 3.2. Influential Operating Parameters on Carotenoid–Pectin Complexation

Table 2 shows the combined responses (yield of the carotenoid–pectin complex) of the complexation of carotenoid and pectin from tomato pomace. The statistically impactful operating parameters on the complexation process were ranked from the most significant to the least significant effect through Pareto chart analysis in the factorial design. Apart from the independent variables, the Pareto analysis estimates the significant interaction between the variables on the responses [22]. The height of the bars is proportional to the significant level of the effect. The orange bars are the effects that exert a positive effect on the response while the blue bars are otherwise. Any effects exceeding the threshold level of *t*-value are considered as significant, while any effects above the Bonferroni limitation line are known to be extremely significant [23].

**Figure 3:**
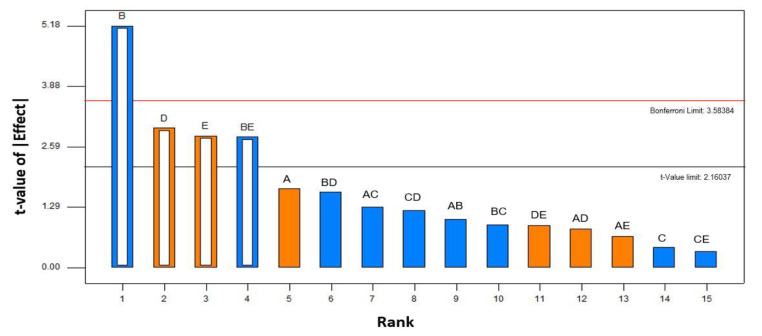
Pareto chart analysis of yield of carotenoid–pectin complexes extracted from tomato pomace. A: pH, B: solid loading, C: temperature; D: stirring speed; E: stirring time; orange bar = positive effect, blue bar = negative effect; empty bar (hierarchical significant factor), full bar (insignificant factor). The standardized effects were at a 95% of confidence interval.

The significance level of the tested operating parameters of the complexation process is shown in Figure 3. Based on the Pareto ranking, solid loading (B) was found to be the most significant variable as its effect has exceeded the Bonferroni limit. Other than solid loading, the effects of stirring speed (D) and stirring period (E) can be considered as the major contributing factors in the complexation process. Thus, these three operating parameters can be suitably selected as the most impactful variables in the complexation phenomenon. The selected operating parameters were further confirmed through ANOVA and the contribution percentage of each operating parameter was calculated. Table 3 shows the combined results of ANOVA and the effect lists on the response. The ANOVA results confirmed that solid loading, stirring speed, and stirring period are the significant factors contributing more than 10% of effect on the complexation phenomenon. Likewise, the second-order interaction factors between solid loading and stirring time also negatively affected the complex formation.

### 3.3. Carotenoid–Pectin Complexation on Tomato Pomace

The antioxidants recovered from food processing by-products find applications in foods [24] and cosmetics [25] whereas the complexation with other ingredients like dietary fiber is a critical factor for their successful implementation. The physical properties and the matrix integrity of the processed tomatoes may differ from other sources of carotenoids. The processed tomatoes are composed of the disintegrated pericarp cells with different levels of fluid content mainly controlled by pectin [26]. Hence, the effects exerted by each variable on the carotenoid–pectin complex collectively and the amount of carotenoid in the complex may vary due to the processing effect. The second-order polynomial equations for the carotenoid–pectin complex and carotenoid content responses were fitted using multiple regression analysis. The empirical relationship between the respective responses and the variables was described by quadratic polynomial equations (coded level) accounting for the significant factors: stirring period (A), stirring speed (B), and solid loading (C), as shown in Equations (2) and (3):

Carotenoid–pectin complex = 1436.98 − 1592.26A − 33.3B − 82C − 25.43AB + 209.56AC + 183.91BC + 1428.93A^2^ − 610.52B^2^ + 46.54C^2^ (2)

Carotenoid = 2.99 − 0.61A − 1.2B + 2.58C − 0.91AB − 1.3AC − 0.41BC + 3.74A^2^ − 3.55B^2^ + 1.23C^2^ (3)

**Figure 4:**
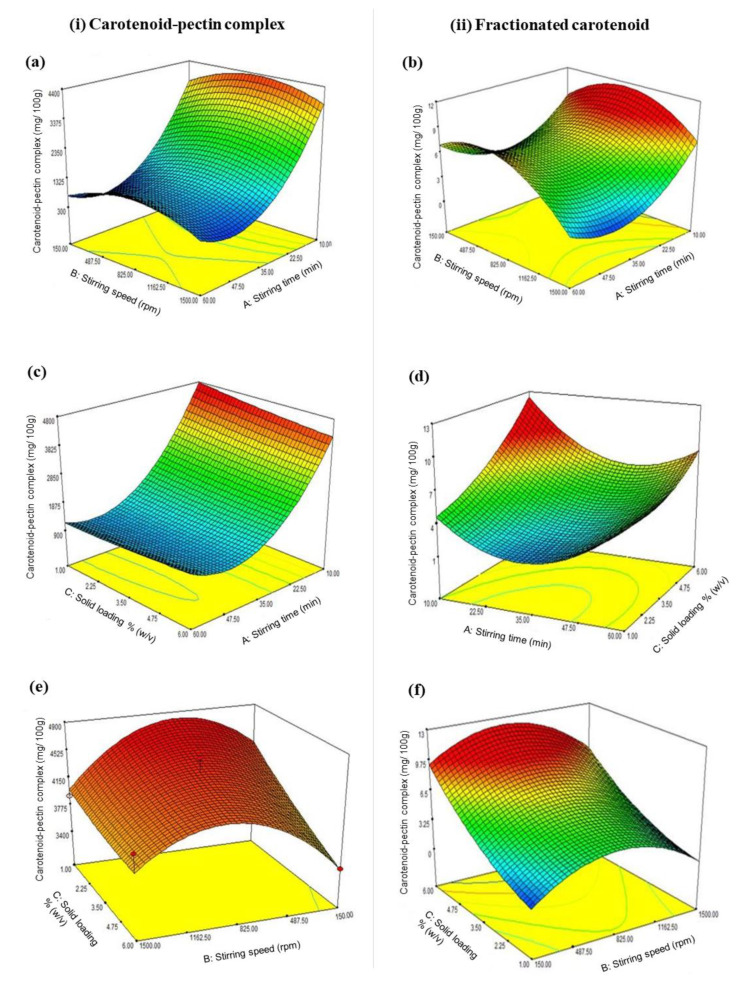
3D response surface plots showing the combined effects of (**a** and **b**) stirring speed and stirring time, (**c** and **d**) solid loading and stirring time, and (**e** and **f**) solid loading and stirring speed, on the yields of (i) carotenoid-pectin complex and (ii) fractionated carotenoid.

The ANOVA results of the fitted quadratic polynomial models of both responses are given in Table 4. The *R*^2^ value of the model for recovery of the carotenoid fraction was lower than that of the model for the recovery of the carotenoid–pectin complex, but the value was within the acceptable range [16,27]. Figure 4 shows the interaction effects of the variables in 3D surface plots for the maximum recovery of both carotenoid–pectin complex and fractionated carotenoids. The desirability function of the model (D) was 0.965, which satisfies the target for the maximum recoveries of the carotenoid–pectin complex as well as the carotenoid fraction from the complex. An increase in the recovery of the carotenoid–pectin complex was contributed by the shorter duration of stirring and the mid-range level of stirring speed (see Figure 4a). For the maximum recovery of the fractionated carotenoids, the interactive effects between stirring speed and stirring time (Figure 4b) indicated that the recovery of the carotenoid fraction increased as the stirring time was shortened, and at the middle range of stirring speed. An increase in stirring time was desirable for a better recovery of the carotenoid–pectin complex only when the loaded amount of solid sample was reduced (see Figure 4c). Besides, an increasing amount of the loaded solid sample along with a shorter duration of stirring were favorable for an improved recovery of carotenoid fraction from the complex (Figure 4d). Figure 4e shows that the maximum recovery of the carotenoid–pectin complex could be best achieved at the middle ranges of both stirring speed and solid loading. The model in Figure 4f demonstrates that the maximum recovery of the carotenoids entrapped in the complex is possibly achieved by increasing the solid loading but maintaining the stirring speed in the intermediate range.

The proposed optimum extraction conditions were at 851 rpm of stirring speed for 10 min and with 4.69% of solid loading. Based on the models, the predicted values for the maximum recoveries of the carotenoid fraction and carotenoid–pectin complex were 9.43 mg/100 g and 4332.32 mg/100 g, respectively. From the experiments, the yields of carotenoids and the carotenoid–pectin complex were 8.53 ± 0.79 mg/100 g and 4356.99 ± 239 mg/100 g, respectively. Both responses exhibited insignificant differences with the predicted values (*p* > 0.05). Hence, the models were validated to be adequate and reliable.

### 3.4. Comparison of Extraction Conditions

The optimum extraction conditions for recovering carotenoid-pectin complex from pink guava decanter and tomato pomace are presented in Table 5. The optimal extraction conditions for each agro-waste is known to be different due to variations in the pre-treatment, size of particles, firmness of matrix, and concentration of carotenoid in the solid matrix. For instance, the pink guava decanter [14] required a greater stirring effect and a lower solid loading than those for the tomato pomace. It is worth noting that the pink guava decanter undergoes several stages of processing (i.e., cutting, crushing, refining, and sieving), hence, the decanter waste has less structural integrity than that of the minimally processed tomato pomace (i.e., the softened form of fruit). A low solid loading with a vigorous stirring can release the maximum amounts of carotenoid and pectin from the plant matter. However, the tomato pomace required a high solid loading as the blended fruit was not extensively disintegrated. The dynamics in the osmotic state of the tomato pomace tissue may also hinder the release of carotenoids and pectin to the solvent; a higher amount sample stirred in mid-range of stirring speed for a short time could best promote the complexation process between carotenoid and pectin from tomato pomace.

### 3.5. Comparison of Carotenoid–Pectin Complexation Method with Conventional Solvent Extraction Method

The efficiency of extraction methods was compared in terms of yield and antioxidant properties of carotenoid–pectin complex. Table 6 shows a comparison of carotenoid yield obtained from tomato pomace by using the complexation method and conventional solvent extraction method. The complexation method was accomplished in a single step with a minimum amount of organic solvent and a shorter extraction time. In contrast, the conventional solvent extraction method involved a large volume of organic solvents and a long duration of extraction (i.e., overnight). In a single extraction process, the complexation method yielded a comparable amount of carotenoid concentration and did not require multiple cycles of extractions involving various types of organic solvents.

**Figure 5:**
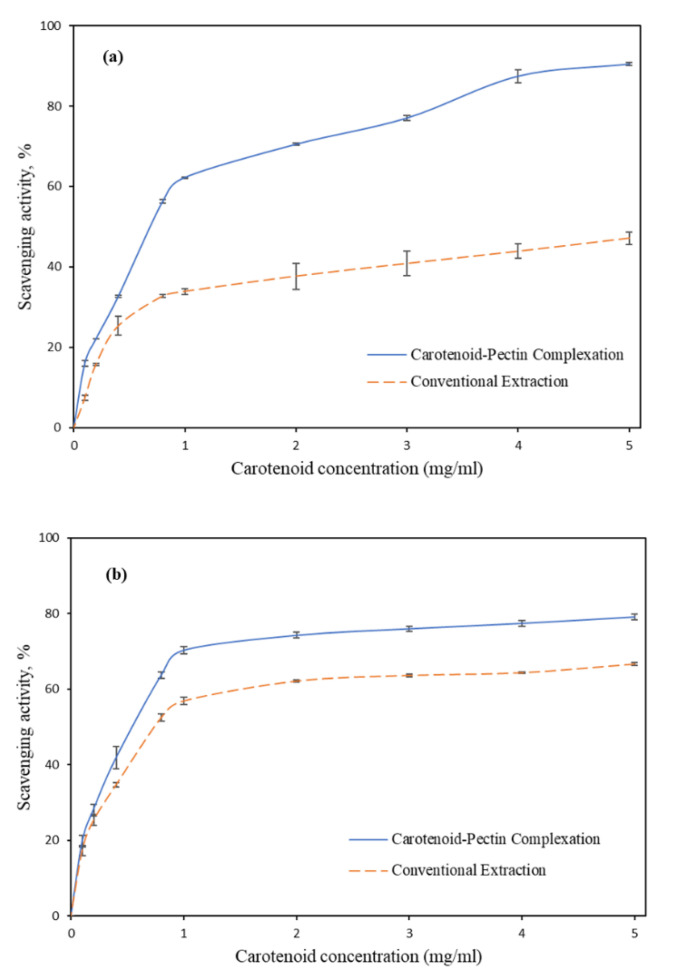
Scavenging activities of (**a**) DPPH and (**b**) ABTS assays on the samples extracted from tomato pomace.

The antioxidant level of carotenoid fractions recovered from both methods was tested using DPPH and ABTS assays. The results are presented in Figure 5. Overall, the extracts resulting from the complexation method contained a relatively higher antioxidant level than that from the solvent extraction method. It is worth emphasizing that the solvent extraction method often requires a round of sample drying prior to the extraction protocols. This is due to the water-immiscible property of organic solvents like hexane for carotenoid extraction. The non-polar solvent possesses poor penetrability in the wet plant matrix. Hence, the extraction efficiencies were enhanced by pre-treating the sample by freezing or oven drying [27,28]. Various scientific findings highlighted that a noticeable loss in the antioxidant properties of plant samples was caused by the drying effect [29,30]. The presence of multiple conjugated double bonds in the carotenoid structure rendered the carotenoid to be sensitive to heat and dehydration [31]. Hence, the antioxidant capacity in the dried sample was lower than that of the wet sample. Besides, the pectin in the recovered complex played a significant role in protecting the carotenoid pigments entrapped in the complex. Pectin in the complex minimizes the external exposure of the carotenoid pigment, thus preserving the antioxidant capacity of the compound.

Figure 5a,b shows the scavenging activities of the tomato pomace’s extract. The carotenoids recovered using the solvent extraction method exhibited 47.14 ± 2.68% and 66.62 ± 0.767% (IC_50_ = 0.75 mg/mL) of the scavenging activities in the DPPH and ABTS assays, respectively, at 5 mg/mL concentration. On the other hand, IC_50_ values of the extract recovered using the carotenoid–pectin complexation method scavenged 90.4 ± 0.61% (IC_50_ = 0.75 mg/mL) and 79.07 ± 0.76% (IC_50_ = 0.55 mg/mL) of free radicals in the DPPH and ABTS assays, respectively, at 5 mg/mL concentration. Hence, the antioxidant levels of carotenoid extracted by complexation were 47.7% and 15.75% higher than that by conventional solvent extraction, as determined from DPPH and ABTS assays, respectively. The low antioxidant level of carotenoid obtained using solvent extraction may be attributed to the drying steps in sample preparation [15]. Based on the results reported for the yield and antioxidant properties of carotenoids, it is evident that the carotenoid–pectin complexation method can be a good substitute to the conventional extraction method. Pectin as obtained from the extraction process can be a secondary bioactive product to be commercialized along with carotenoid. Depending on the end-use application, the carotenoid–pectin complex can be directly used without the need for fractionation with the organic solvent.

## 4. Conclusions

The versatility of the carotenoid–pectin complexation extraction method on tomato pomace was demonstrated. With a simple extraction protocol mainly relying on water solvent, this green extraction process has the lowest levels of impact to the processing cost and the environment. The fractionated carotenoid and pectin were structurally confirmed through HPLC and FTIR analyses. Favorably, this complexation method was able to selectively extract the carotenoid from a plant matrix in the form of a pectin-containing complex. The carotenoid fractions recovered from the complexes had a high purity level (92%). Among the operating parameters, the influential variables on the carotenoid–pectin complexation were solid loading, stirring speed, and stirring duration. Most of the regression models satisfactorily predicted the yields of the carotenoid–pectin complexes and fractionated carotenoids. The carotenoid–pectin complexation method was compared with the conventional method in terms of the extraction yield and the ability for scavenging free radicals. The fractionated carotenoids from the complexation method possessed a higher level of antioxidant properties than those obtained from the conventional extraction methods. Overall, the carotenoid–pectin complexation process is a green extraction method that can be suitably applied to tomato pomace and other agro-waste sources rich in both pectin and carotenoids.

## Figures and Tables

**Table 1:** Range limit of variables used in the two-level half factorial design.

Variable	Notation	Unit	Ranges of Variable		
			Low (−1)	Intermediate (0)	High (1)
pH	A		1	5	9
Solid loading	B	%	1	3.5	6
Temperature	C	°C	25	45	65
Stirring speed	D	rpm	150	825	1500
Stirring period	E	min	10	35	60

**Table 2:** Factorial design showing the variables (coded) and the response for carotenoid–pectin complexes extracted from tomato pomace.

Standard No.	Independent Variables					Carotenoid–Pectin Complex (mg/100 g of Wet Sample)
	A	B	C	D	E	Tomato Pomace
1	−1	−1	−1	−1	1	3259
2	1	−1	−1	−1	−1	1840
3	−1	1	−1	−1	−1	823
4	1	1	−1	−1	1	1280
5	−1	−1	1	−1	1	2459
6	1	−1	1	−1	−1	6249
7	−1	1	1	−1	−1	533
8	1	1	1	−1	1	360
9	−1	−1	−1	1	1	3039
10	1	−1	−1	1	−1	11,953
11	−1	1	−1	1	−1	1943
12	1	1	−1	1	1	3090
13	−1	−1	1	1	1	7900
14	1	−1	1	1	−1	4939
15	−1	1	1	1	−1	770
16	1	1	1	1	1	1347
17 ^a^	0	0	0	0	0	3947
18 ^a^	0	0	0	0	0	3651
19 ^a^	0	0	0	0	0	4432

Note: ^a^: the central points (intermediate values) of the experimental runs.

**Table 3:** ANOVA analysis and contribution percentage of the variables for extraction of carotenoid–pectin complexes from tomato pomace.

Source	Sum of Squares	DF	Mean Square	*F* Value	*p* Value	Significance	Contr. (%)
**Tomato pomace**							
Model	1.19 × 10^8^	4	2.98 × 10^7^	12.88	0.0002	Significant	−
B	6.20 × 10^7^	1	6.20 × 10^7^	26.8	0.0002	Significant	41.13
D	2.07 × 10^7^	1	2.07 × 10^7^	8.93	0.0105	Significant	13.70
E	1.84 × 10^7^	1	1.84 × 10^7^	7.94	0.0145	Significant	12.19
BE	1.81 × 10^6^	1	1.81 × 10^6^	7.83	0.0151	Significant	12.02
Residual	3.01 × 10^7^	13	2.31 × 10^6^				
Lack of Fit	2.98 × 10^7^	11	2.71 × 10^6^	17.4	0.0556	Insignificant	
Pure Error	3.11 × 10^5^	2	3.11 × 10^5^				
Corr. Total	1.51 × 10^8^	18					

Note: Corr. Total: corrected total; Contr.: contribution; DF: degree of freedom.

**Table 4:** ANOVA results of the fitted quadratic polynomial models of yields of carotenoid-pectin complex and fractionated carotenoid.

Source	SS	DF	MS	*F* Value	*p* Value
**Carotenoid–pectin Complex from Tomato Pomace (*R*^2^ = 0.9281)**					
Model	3.314 × 10^7^	9	3.682 × 10^6^	14.34	0.0001
Lack of fit	2.009 × 10^6^	5	4.019 × 10^5^	3.61	0.0928
Pure error	5.572 × 10^5^	5	1.114 × 10^5^		
**Carotenoid Fraction from Tomato Pomace (*R*^2^ = 0.8687)**					
Model	163.84	9	18.70	7.35	0.0022
Lack of fit	19.91	5	3.98	3.60	0.0932
Pure error	5.54	5	1.11		

Note: SS: sum of squares; DF: degree of freedom; MS: mean square.

**Table 5:** Optimum extraction conditions of carotenoid–pectin complex for recovery of carotenoid and pectin from different agro-wastes.

Agro-Waste	Stirring Time (min)	Stirring Speed (rpm)	Solid Loading (%, w/v)	Reference
Decanter	34	1098	1.00	[14]
Tomato pomace	10	851	4.69	This work

**Table 6:** Extraction yield of carotenoid from carotenoid–pectin complexation and solvent extraction.

Sample	Carotenoid–Pectin Complexation		Solvent Extraction	
	Carotenoid (mg/100 g Wet Sample)	TNE	Carotenoid (mg/100 g Wet Sample)	TNE
Tomato pomace	9.37 ± 1.34	1	10.81 ± 0.57	2

Note: ± standard deviation; TNE: total number of extractions.
